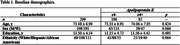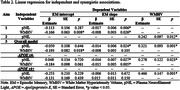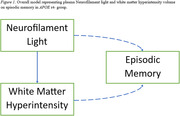# Association of plasma neurofilament light and white matter hyperintensity on memory: Differential findings across Apolipoprotein E risk groups in normal aging

**DOI:** 10.1002/alz.093216

**Published:** 2025-01-09

**Authors:** Mae Yue So, Pauline Maillard, Lee‐Way Jin, Sarah Tomaszewski Farias, Charles Decarli, Shraddha Sapkota

**Affiliations:** ^1^ University of California, Davis, Davis, CA USA

## Abstract

**Background:**

Neurocognitive trajectories in normal aging are a result of complex and synergistic associations of multiple risk domains. Blood‐based, neuroimaging, and genetic biomarkers all play fundamental roles that span decades leading to cognitive decline. We study all three risk domains on memory using an ethnoracially diverse cohort. Specifically, we examine (1) independent associations of plasma Neurofilament Light (pNfL) levels and white matter hyperintensity volume (WMHV) on episodic memory (EM), (2) pNfL levels on WMHV, and (3) synergistic model with pNfL and WMHV to predict EM, independently and as stratified by Apolipoprotein E (APOE) risk groups.

**Method:**

We used an ethnically diverse cohort of cognitively normal (CN) older adults from the University of California, Davis‐Alzheimer’s Research Center (n=299, Table 1). EM was measured using the Spanish and English Neuropsychological Assessment Scales across eleven waves. WMHV and pNfL levels were obtained at baseline. Statistical analyses included a latent growth modeling to establish latent EM growth model, and regression analyses to test independent associations and stratification with APOE. APOE genotype risk was examined as ε4‐/ε4+. Age was centered at 75 years, and sex and education were included as covariates.

**Result:**

First, higher pNfL level was associated with steeper EM decline (Table 2). Second, higher WMHV was associated with lower EM performance and steeper EM decline. Third, higher pNfL level was associated with higher baseline WMHV. Fourth, in the combined model, higher pNfL level was associated with steeper EM decline, higher WMHV was associated with lower EM performance, and higher pNfL level was associated with higher baseline WMHV. When stratified by APOE genotype risk, higher pNfL level and higher WMHV were associated with steeper EM decline only in the APOE ε4‐ group. However, higher pNfL level was associated with higher baseline WMHV across APOE ε4‐ and ε4+ groups (Figure 1).

**Conclusion:**

We observed that the association between pNfL and EM, and WMHV and EM are both modified by APOE genotype (Figure 1). However, the relationship between pNfL and WMHV is present regardless of genetic risk. Examining biomarkers across multiple domains may lead to novel and effective clinical interventions in diverse CN populations.